# Disentangling the genetics of sarcopenia: prioritization of *NUDT3* and *KLF5* as genes for lean mass & *HLA-DQB1-AS1* for hand grip strength with the associated enhancing SNPs & a scoring system

**DOI:** 10.1186/s12881-020-0977-6

**Published:** 2020-02-24

**Authors:** Abhishek Narain Singh, Bili Gasman

**Affiliations:** 1 A.I. Virtanen Institute for Molecular Sciences, University of Eastern Finland, Kuopio, Finland; 2 Faculty of Medicine, Bar Ilan University, Safed, Israel

**Keywords:** SNPs, GWAS, eQTL, TADs, TWAS, muscle, SMR, sarcopenia

## Abstract

**Background:**

Sarcopenia is a skeletal muscle disease of clinical importance that occurs commonly in old age and in various disease sub-categories. Widening the scope of knowledge of the genetics of muscle mass and strength is important because it may allow to identify patients with an increased risk to develop a specific musculoskeletal disease or condition such as sarcopenia based on genetic markers.

**Methods:**

We used bioinformatics tools to identify gene loci responsible for regulating muscle strength and lean mass, which can then be a target for downstream lab experimentation validation. Single nuclear polymorphisms (SNPs) associated with various disease traits of muscles and specific genes were chosen according to their muscle phenotype association *p*-value, as traditionally done in Genome Wide Association Studies, GWAS. We’ve developed and applied a combination of expression quantitative trait loci (eQTLs) and GWAS summary information, to prioritize causative SNP and point out the unique genes associated in the tissues of interest (muscle).

**Results:**

We found *NUDT3* and *KLF5* for lean mass and *HLA-DQB1-AS1* for hand grip strength as candidate genes to target for these phenotypes. The associated regulatory SNPs are rs464553, rs1028883 and rs3129753 respectively.

**Conclusion:**

Transcriptome Wide Association Studies, TWAS, approaches of combining GWAS and eQTL summary statistics proved helpful in statistically prioritizing genes and their associated SNPs for the disease phenotype of study, in this case, Sarcopenia. Potentially regulatory SNPs associated with these genes, and the genes further prioritized by a scoring system, can be then wet lab verified, depending on the phenotype it is hypothesized to affect.

## Background

Many diseases known to man originate from more than one genetic locus. Sarcopenia for example is multifactorial [1] degenerative loss of skeletal muscle mass, a condition that might pose a great risk for the aging world population. Since 2006, GWAS have allowed us to trace the multiple genetic factors for various traits using statistical tools that can lead to a more effective research of specific loci of interest [2]. The data produced by these studies, which now rank in the thousands, is available online so further downstream research can be conducted, and new results can be incorporated. This is indeed valuable, since musculoskeletal diseases are one of the leading causes of disability in the world [3]; treatment of these diseases costs the world medical industry around 125 billion dollars annually [3].

In this paper, we present the combination of summary level data from GWAS and publicly available eQTLs such as those from studies by GTEx [4] and Westra et al. [5]. Based on available data and our approach of combining phenotype-associated SNPs (Single Nucleotide Polymorphism) and tissue-relevant gene-associated SNPs TADs (topologically associated domains) were plotted at the regions of interest. A TAD is a self-interacting genomic region, meaning that DNA sequences within a TAD physically interact with each other more frequently than with sequences outside the TAD [6].

We developed and applied a combination of expression quantitative trait loci (eQTLs) and GWAS summary information, to prioritize causative SNP and point out the unique genes associated in the tissues of interest (muscle).

## Method

The results of GWAS for lean mass (LM) and hand grip strength (HG) were published in studies by Karasik et al. [7], Zilikens et al. [8], Willems et al. [9] and Tikkanen et al. [10], in various large human populations. According to consensus in the literature, we used the genome-wide significance threshold of 5*10^− 8^ to consider SNPs to be associated for a follow-up. The summary of eQTL data was obtained by studies by Westra et al. [5] and by the GTEx [4] consortium. From the study of Westra et al. eQTLs of HSMM (Human skeletal muscle myoblasts) culture were obtains, while the GTEx consortium EQTLs were from human striated muscle samples. With these data sets, we executed “Summary-data-based Mendelian randomization” (SMR) analysis using the method as proposed by Zhu et al. [11] and utilizing the “SMR tool” program, version 0.710. We chose not to investigate the SNPs for further categorization of pleiotropy or causality, which can be done using the Heidi test [11]. For the case of GTEx eQTL summary data, the execution was done for all tissues, and then we observed for genes which were enriched specifically in skeletal muscle tissue or specifically compared to the aggregate of all other tissue types. For the genes of interest as described in the above method, we went on to plot and examine TADs at the relevant regions in corresponding skeletal muscle tissues such as the psoas (striated) and bladder (smooth muscle) as done by Schmitt et al. [12]

## Results

**Fig. 1:**
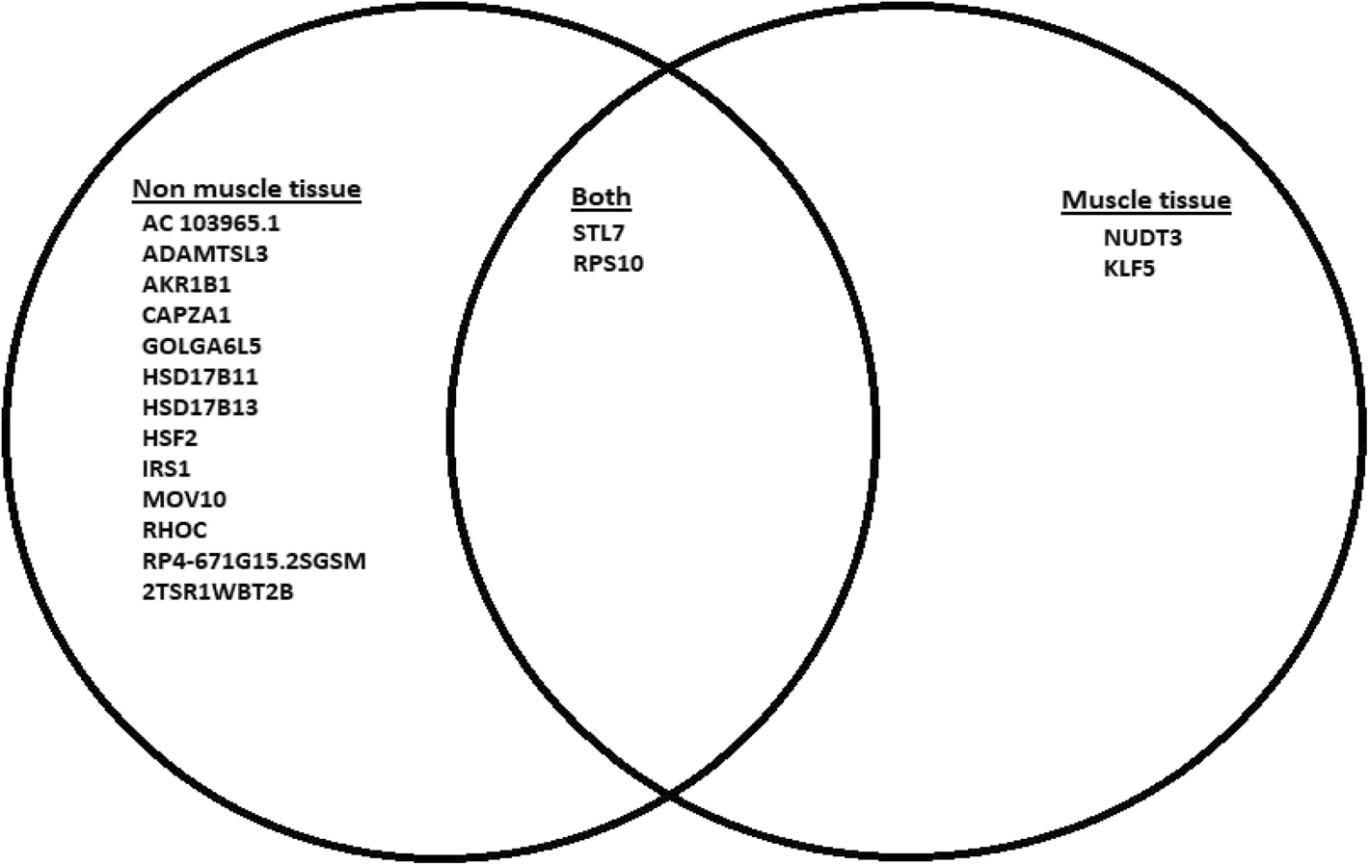
Gene expression based on SMR of GTEx eQTLs and whole-body lean mass GWAS

**Fig. 2:**
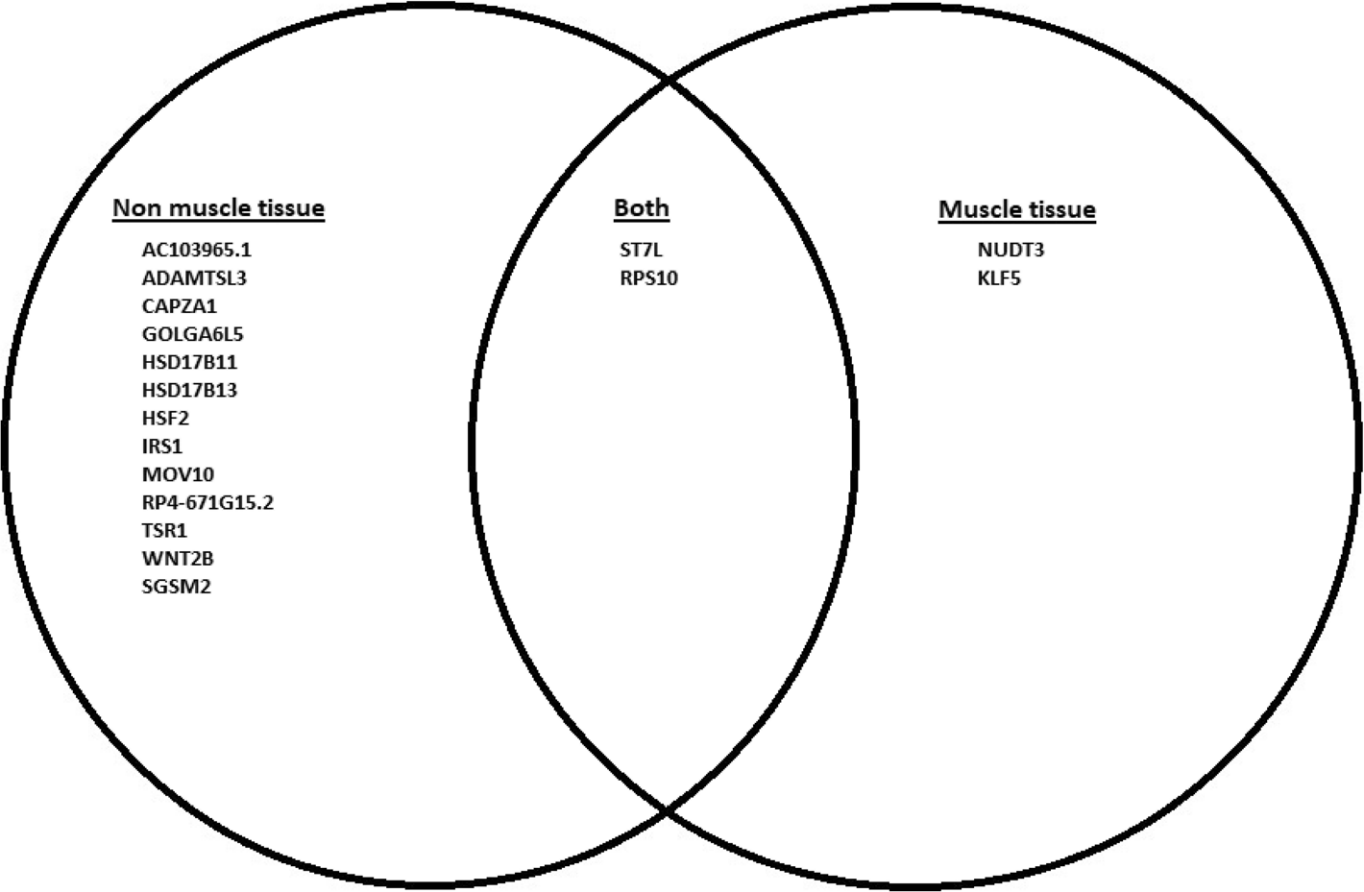
Gene expression based on SMR of GTEx eQTLs and appendicular lean mass GWAS

**Fig. 3:**
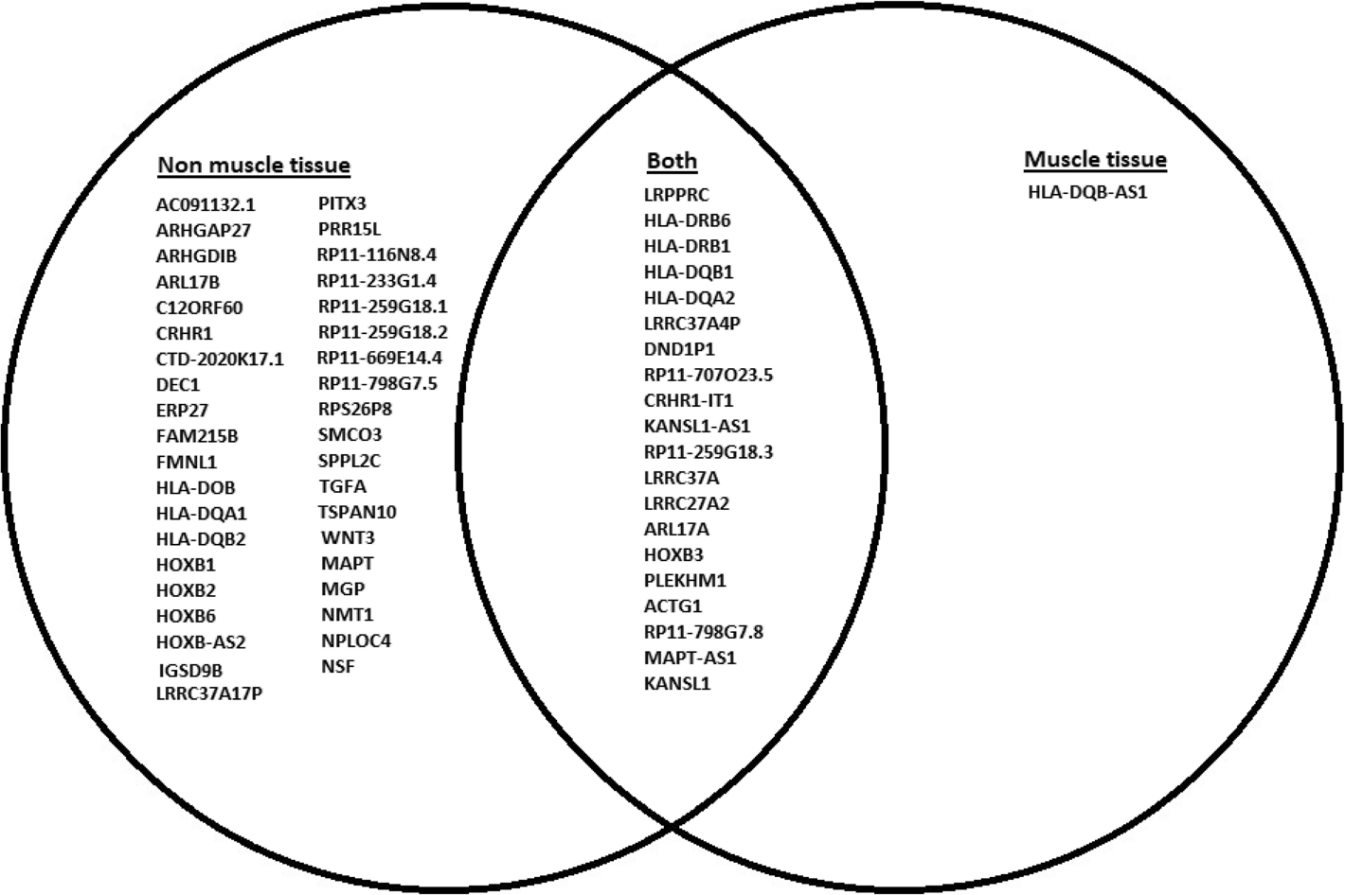
Gene expression based on Handgrip GWAS data and GTEx eQTL enrichment analysis

**Fig. 4:**
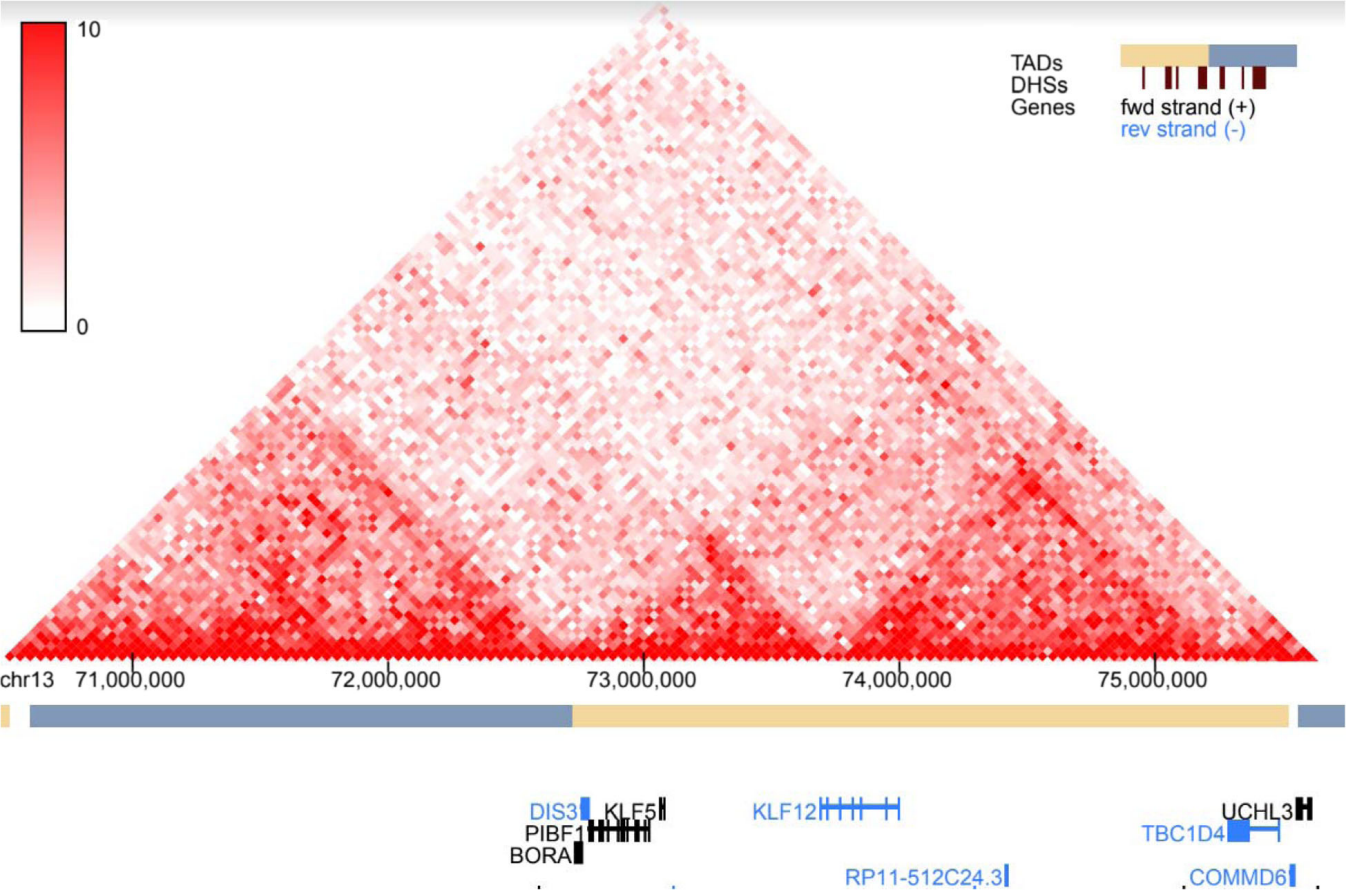
KLF5 expression in chromosome 13 (Bladder muscle)

**Fig. 5:**
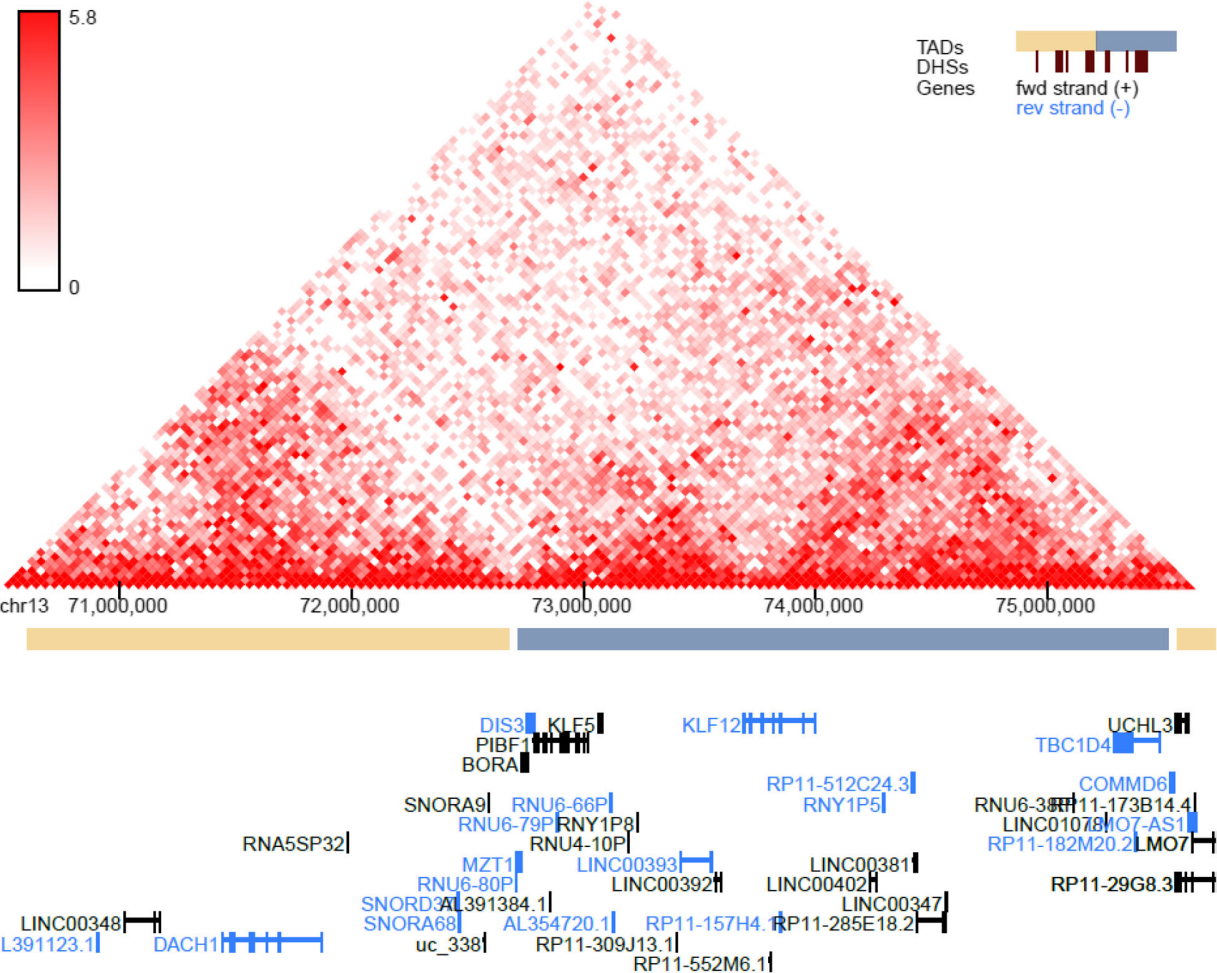
KLF5 expression in chromosome 13 (psoas muscle)

**Fig. 6:**
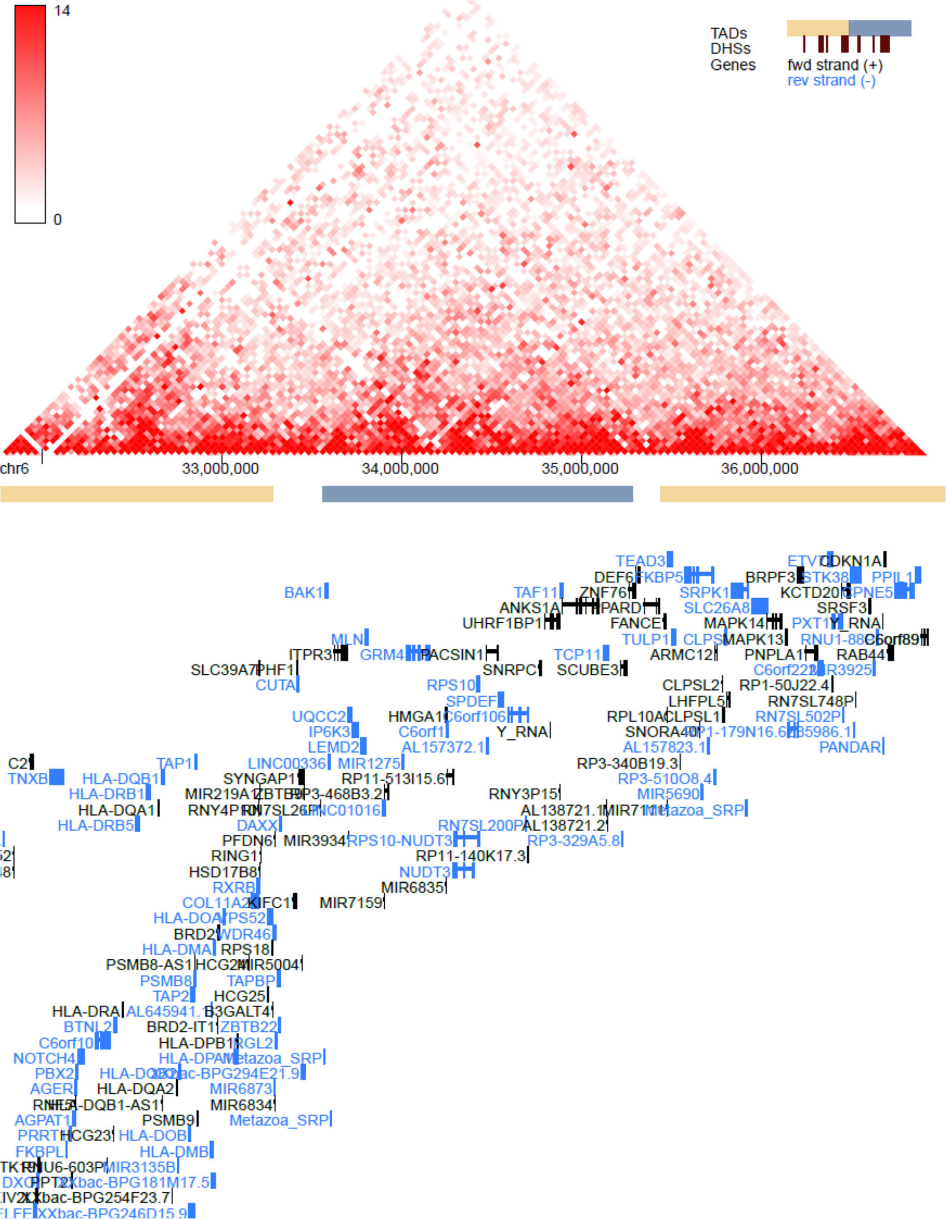
NUDT3 expression in chromosome 6 (bladder muscle)

**Fig. 7:**
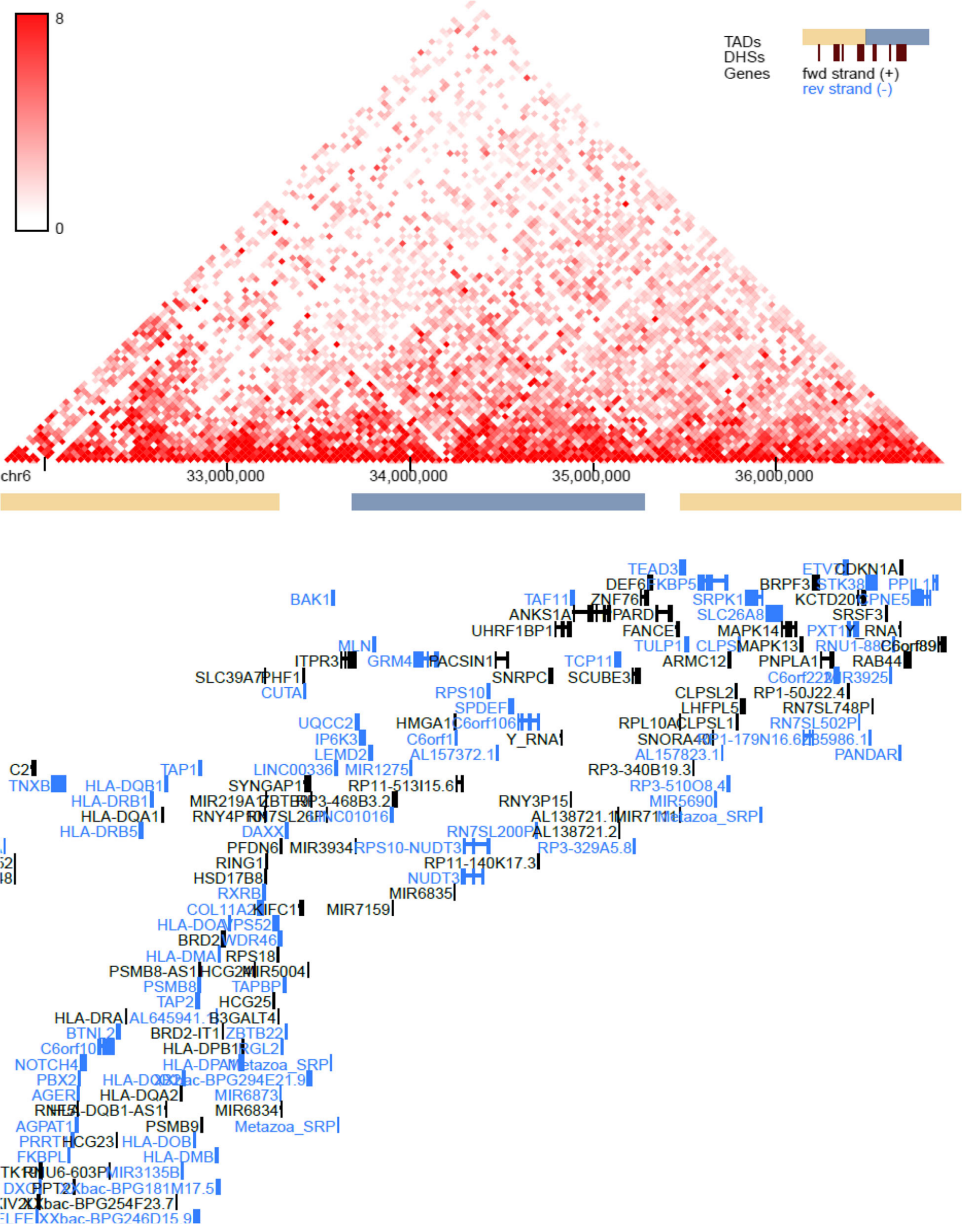
NUDT3 expression in chromosome 6 (psoas muscle)

GTEx tissue analysis found that for lean mass, two genes: *NUDT3* and *KLF5*, were enriched in skeletal muscles (Figs. 1, 2), and they were also found in Westra et al. [5] eQTL analysis for appendicular lean mass (Table 1), although not in whole body in the case of Westra et al. [5] study (Table 2). Venn diagram in Fig. 1 is derived from Tables 3 and 4; Venn diagram in Fig. 2 is derived from Tables 5 and 6. In the GTEx tissue analysis for the hand grip trait, we found one gene, *HLA-DQB1-AS1*, which was specifically enriched in skeletal tissue compared to other tissues (Fig. 3), with the associated SNP as rs3129753. The Venn diagram in Fig. 3 is derived from Tables 7 and 8. Many other genes found to be enriched in skeletal muscle tissues and other tissues in common intersection (Figs. 1, 2 and 3) were also found in Westra eQTL analysis with our GWAS summary dataset. The second priority should be given to the genes found to be enriched in skeletal muscle tissue as well as any other tissue. Clearly, *NUDT3* and *KLF5* are very strong candidate genes for lean mass study, and their associated regulating SNP are rs464553 and rs1028883 respectively. TAD plots for the psoas and bladder tissues (which are skeletal and smooth muscle types, respectively) were plotted (Figs. 4, 5, 6 and 7) where *KLF5* is seen to be present within a FIRE [12] (frequently interacting region) within the TAD of chromosome 13 in bladder (*M.Detrusor)* muscle (Fig. 4).

**Table 1:** Metal SMR of Westra et al. eQTLs and appendicular lean mass GWAS

probe ID	chromosome	Gene	base pair of probe	associated SNP
ENSG00000007341.14	1	*ST7L*	113,122,614	rs1110043
ENSG00000272325.1	6	*NUDT3*	34,307,498	rs464553
ENSG00000124614.9	6	*RPS10*	34,389,566	rs464553
ENSG00000102554.9	13	*KLF5*	73,640,395	rs1028883

**Table 2:** SMR of Westra et al. eQTLs and whole-body lean mass GWAS

probe ID	chromosome	Gene	base pair of probe	associated SNP
ILMN_1659926	1	*ST7L*	112,886,029	rs1110043
ILMN_2410113	1	*ST7L*	112,900,108	rs7522288
ILMN_1725700	1	*MOV10*	113,044,669	rs2999156
ILMN_1673305	1	*RHOC*	113,045,326	rs2999156
ILMN_2313730	1	*RHOC*	113,045,386	rs2999156
ILMN_1701731	7	*AKR1B1*	133,777,993	rs7795758

**Table 3:** SMR of GTEx eQTLs and lean mass GWAS, results for all tissue types

probe ID	chromosome	Gene	base pair of probe	associated SNP
ENSG00000007341.14	1	*ST7L*	113,122,614	rs1110043
ENSG00000116489.8	1	*CAPZA1*	113,188,330	rs1110043
ENSG00000169047.5	2	*IRS1*	227,630,254	rs2943656
ENSG00000198189.6	4	*HSD17B11*	88,285,150	rs9991501
ENSG00000124614.9	6	*RPS10*	34,389,566	rs464553
ENSG00000025156.8	6	*HSF2*	122,737,477	rs1991642
ENSG00000007341.14	1	*ST7L*	113,122,614	rs1110043
ENSG00000007341.14	1	*ST7L*	113,122,614	rs1110043
ENSG00000007341.14	1	*ST7L*	113,122,614	rs1110043
ENSG00000007341.14	1	*ST7L*	113,122,614	rs1110043
ENSG00000007341.14	1	*ST7L*	113,122,614	rs1110043
ENSG00000116489.8	1	*CAPZA1*	113,188,330	rs1110043
ENSG00000170509.7	4	*HSD17B13*	88,234,499	rs9991501
ENSG00000198189.6	4	*HSD17B11*	88,285,150	rs9991501

**Table 4:** SMR of GTEx eQTLs and lean mass GWAS, results for skeletal muscles

probe ID	chromosome	Gene	base pair of probe	associated SNP
ENSG00000007341.14	1	*ST7L*	113,122,614	rs1110043
ENSG00000272325.1	6	*NUDT3*	34,307,498	rs464553
ENSG00000124614.9	6	*RPS10*	34,389,566	rs464553
ENSG00000102554.9	13	*KLF5*	73,640,395	rs1028883

**Table 5:** Metal SMR of GTEx eQTLs and appendicular lean mass GWAS

probe ID	chromosome	Gene	base pair of probe	associated SNP
ENSG00000007341.14	1	*ST7L*	113,122,614	rs1110043
ENSG00000116489.8	1	*CAPZA1*	113,188,330	rs1110043
ENSG00000169047.5	2	*IRS1*	227,630,254	rs2943656
ENSG00000198189.6	4	*HSD17B11*	88,285,150	rs9991501
ENSG00000124614.9	6	*RPS10*	34,389,566	rs464553
ENSG00000025156.8	6	*HSF2*	122,737,477	rs1991642
ENSG00000007341.14	1	*ST7L*	113,122,614	rs1110043
ENSG00000007341.14	1	*ST7L*	113,122,614	rs1110043
ENSG00000007341.14	1	*ST7L*	113,122,614	rs1110043
ENSG00000007341.14	1	*ST7L*	113,122,614	rs1110043
ENSG00000007341.14	1	*ST7L*	113,122,614	rs1110043
ENSG00000116489.8	1	*CAPZA1*	113,188,330	rs1110043
ENSG00000170509.7	4	*HSD17B13*	88,234,499	rs9991501
ENSG00000198189.6	4	*HSD17B11*	88,285,150	rs9991501
ENSG00000007341.14	1	*ST7L*	113,122,614	rs6537748
ENSG00000273483.1	1	*RP4-671G15.2*	113,060,742	rs1110043
ENSG00000134245.13	1	*WNT2B*	113,040,975	rs1110043
ENSG00000273483.1	1	*RP4-671G15.2*	113,060,742	rs1110043
ENSG00000007341.14	1	*ST7L*	113,122,614	rs7522288

**Table 6:** SMR of GTEx eQTLs and appendicular lean mass GWAS, results for skeletal muscle

probe ID	chromosome	Gene	base pair of probe	associated SNP
ENSG00000007341.14	1	*ST7L*	113,122,614	rs1110043
ENSG00000272325.1	6	*NUDT3*	34,307,498	rs464553
ENSG00000124614.9	6	*RPS10*	34,389,566	rs464553
ENSG00000102554.9	13	*KLF5*	73,640,395	rs1028883

**Table 7:** SMR of GTEx skeletal muscle eQTLs and handgrip GWAS

probe ID	chromosome	Gene	base pair of probe	associated SNP
ENSG00000138095.14	2	*LRPPRC*	44,168,395	rs6544736
ENSG00000229391.3	6	*HLA-DRB6*	32,524,144	rs3129751
ENSG00000196126.6	6	*HLA-DRB1*	32,552,085	rs521539
ENSG00000223534.1	6	*HLA-DQB1-AS1*	32,628,319	rs3129753
ENSG00000179344.12	6	*HLA-DQB1*	32,631,702	rs521539
ENSG00000237541.3	6	*HLA-DQA2*	32,712,055	rs6931277
ENSG00000225190.4	17	*PLEKHM1*	43,540,690	rs62055516
ENSG00000214425.2	17	*LRRC37A4P*	43,603,193	rs389978
ENSG00000266918.1	17	*RP11-798G7.8*	43,610,073	rs2668668
ENSG00000264070.1	17	*DND1P1*	43,663,766	rs2266497
ENSG00000263503.1	17	*RP11-707O23.5*	43,678,970	rs2266497
ENSG00000204650.9	17	*CRHR1-IT1*	43,711,638	rs62061802
ENSG00000264589.1	17	*MAPT-AS1*	43,946,991	rs2668668
ENSG00000120071.8	17	*KANSL1*	44,205,007	rs62073098
ENSG00000214401.4	17	*KANSL1-AS1*	44,272,515	rs55974014
ENSG00000262539.1	17	*RP11-259G18.3*	44,337,444	rs56364632
ENSG00000176681.10	17	*LRRC37A*	44,392,629	rs17660294
ENSG00000238083.3	17	*LRRC37A2*	44,609,846	rs439558
ENSG00000185829.11	17	*ARL17A*	44,625,578	rs3865315
ENSG00000120093.7	17	*HOXB3*	46,646,933	rs890432
ENSG00000184009.5	17	*ACTG1*	79,483,935	rs6565586

**Table 8:** GTEx Handgrip GWAS data with eQTL aggregated SMR

probe ID	chromosome	Gene	base pair of probe	associated SNP
ENSG00000138095.14	2	*LRPPRC*	44,168,395	rs6544736
ENSG00000229391.3	6	*HLA-DRB6*	32,524,144	rs3129751
ENSG00000196126.6	6	*HLA-DRB1*	32,552,085	rs521539
ENSG00000223534.1	6	*HLA-DQB1-AS1*	32,628,319	rs3129753
ENSG00000179344.12	6	*HLA-DQB1*	32,631,702	rs521539
ENSG00000237541.3	6	*HLA-DQA2*	32,712,055	rs6931277
ENSG00000225190.4	17	*PLEKHM1*	43,540,690	rs62055516
ENSG00000214425.2	17	*LRRC37A4P*	43,603,193	rs389978
ENSG00000266918.1	17	*RP11-798G7.8*	43,610,073	rs2668668
ENSG00000264070.1	17	*DND1P1*	43,663,766	rs2266497
ENSG00000263503.1	17	*RP11-707O23.5*	43,678,970	rs2266497
ENSG00000204650.9	17	*CRHR1-IT1*	43,711,638	rs62061802
ENSG00000264589.1	17	*MAPT-AS1*	43,946,991	rs2668668
ENSG00000120071.8	17	*KANSL1*	44,205,007	rs62073098
ENSG00000214401.4	17	*KANSL1-AS1*	44,272,515	rs55974014
ENSG00000262539.1	17	*RP11-259G18.3*	44,337,444	rs56364632
ENSG00000176681.10	17	*LRRC37A*	44,392,629	rs17660294
ENSG00000238083.3	17	*LRRC37A2*	44,609,846	rs439558
ENSG00000185829.11	17	*ARL17A*	44,625,578	rs3865315
ENSG00000120093.7	17	*HOXB3*	46,646,933	rs890432
ENSG00000184009.5	17	*ACTG1*	79,483,935	rs6565586

## Discussion

Apart from the combination of GWAS and eQTL summaries for the tissue of interest and searching for exclusivity of gene enhancement and the presence of SNP regulation near TAD boundaries, we also incorporated a novel scoring system to prioritize genes for functional validation of our results. Functional validation is a slow and costly process. Validation can take months and even years to complete without a promise of a positive result, hence a scoring system is vital for scrutinizing and grading our results to asses which gene might have an effect on muscle health. The process of functional validation is vital for a few reasons. For one, relying on TADs has its limitations. Chromosomes separate active and inactive chromatin into compartments A and B, respectively where compartment A correlates with high gene expression, active histone marks, and early replication timing, whereas the compartment B replicates late, is enriched with repressive histone modifications and has low gene expression. Compartments can be further subdivided into megabase-sized genomic regions known as topologically associating domains (TADs) [13, 14]. the function of TADs is not fully understood yet, although disrupting the TADs e.g. because of SNPs or InDels (insertions deletions) may result in the establishment of novel inter-TAD interactions. These have been shown to be associated with misexpression of Hox genes [15], up-regulation of proto-oncogenes [16], and developmental disorders [14]. Furthermore, functional validation might also allow us to identify drugs that affect muscle in ways unknown before and therefore to reposition existing drugs to other uses, in accordance to their newly found target such as targeted gene therapy as discussed in context of next generation sequencing in drug development [17]. This serves two purposes, first is validation of the scoring system itself as an algorithm for GWAS result validation, and the more important one is validation of new targets for further research and potentially, repositioning of existing drugs.

Our approach has its limitations and requires validation, as one can observe from the results. In spite the fact that TADs that were plotted by our approach of combining phenotype-associated SNPs and tissue-relevant gene-associated SNPs show that the genes of interest are located within a frequently interacting region, the rest of the data regarding these genes doesn’t support our hypothesis that they in fact have an effect on muscle health. *NUDT3* (Nudix Hydrolase 3) for example, codes for the Nudix protein which act as homeostatic checkpoints at important stages in nucleoside phosphate metabolic pathways, guarding against elevated levels of potentially dangerous intermediates [18]. GWAS associate *RSP-NUDT3* readthrough to BMI with a *p*-value of 4*10^− 12^ [19]. The Malacards database also associates *NUDT3* with hyperinsulinism and obesity in specific populations [20]. *KLF5* (Kruppel Like Factor 5), encodes a member of the Kruppel-like factor subfamily of zinc finger proteins. The encoded protein is a transcriptional activator that binds directly to a specific recognition motif in the promoters of target genes. This protein acts downstream of multiple different signaling pathways and is regulated by post-translational modification [21]. GWAS catalogue doesn’t relate this gene to muscle health phenotypes [19], the same is true for the Malacards [20] database. In contrast, the STRING database [22] finds relation between *NUDT3* and *ACTA2* (Actin Alpha 2) and *GSK3B* (Glycogen Synthase Kinase 3 Beta) which are related to actin production and energy metabolism respectively [21]. *HLA-DQB1-AS1 (HLA-DQB1 Antisense RNA 1)* is an RNA Gene and is affiliated with the lncRNA (Long non-coding RNA) class is related to malignant diseases and doesn’t seem to be associated with muscle wasting disorders according to MalaCards [20]. The above information emphasizes that these genes are not directly related to muscle health, yet they may have some indirect regulatory role in defining it. Functional validation is vital in the process of confirming or debunking the hypothesis that these genes are associated with muscle health. We suggest that the above genes be scrutinized using a scoring system as described below, for prioritizing candidate genes for functional validation which will be done by knocking out the gene in C2C12 mouse myoblast cell, assessing gene expression using RT-qPCR and comparing cell morphology to the morphology wild type C2C12 cells. The scoring system has been briefly stated in the form of abstract [23] earlier and we mention it in detail here. The following constitutes the scoring system proposed for functional validation of our results: Potential genes were obtained from the work of Zillikens et al. [8], Karasik et al. [7] for LM and for HG, Willems et al. [9] and Tikkanen et al. [10]. Genes provided by Karasik et al. Zillikens et al. and Willems et al. were graded as first tier genes, while genes provided by Tikkanen et al. were graded as second tier genes. The reason behind this is that the Tikkanen et al. research was published at the end of the year 2018, while the database was already being collected. The list of SNPs was mined with cis Expression quantitative trait loci analysis (eQTLs) for transcripts within 2 Mb of the SNP position was carried out as described by Zillikens et al. [8]. Other similar datasets were scrutinized, and genes in proximity of SNPs were scaled according to a specifically developed scoring system which utilized the following publicly available databases: Malacards [20], COXPRESdb [24] gene co-expression database, PubMed search engine, Ensembl database [25], the mouse genome informatics database [26], HaploReg [27] and the LDlink [28] database.

The above functional validation method combined with our approach to gene prioritization might help in Identifying new loci responsible for LM or HG, and thus identifying new genetic markers for sarcopenia. This approach may also be used by the pharmaceutical industry to identify targets for new pharmaceutical products or reposition existing drugs in accordance to new data on the activity of these drugs. The greatest hurdle with drug repositioning today is lack of solid databases needed to produce good results [24]. Functional validation of the results presented in this study results can serve as a test to whether our approach to gene prioritization can resolve this problem.

## Conclusions

The current work focused primarily on the combined bioinformatic approaches using GWAS and eQTLs for SMR. The results of exclusivity of the tissues of interest were further classified for their importance based on Venn diagrams and their corresponding TAD plots to look for the TAD boundaries where the associated regulating SNPs could be localized. *NUDT3* and *KLF5* for lean mass and *HLA-DQB1-AS1* for hand grip strength and their associated SNPs (rs464553, rs1028883 and rs3129753) had the highest priority as candidate targets for further study.

One limitation of this study is that the eQTL analysis was not done on trans-association SNPs. Another is the limited knowledge on TAD function.

We propose functional validation by a method of gene knock out in C2C12 mouse myoblast cells to either prove or rebut the effect of prioritized genes on muscle health, thus widening the scope of knowledge on the genetic origins of sarcopenia.

## Data Availability

www.tinyurl.com/abinarain and then navigate to Educational Section and click where its written ‘GWAS eQTL Summary Approach with TADs for Skeletal Muscle work’.

## Abbreviations

eQTL: Expression quantitative trait loci
FIRE: Frequently interacting region
GTEx: Genotype Tissue Expression
GWAS: Genome wide association study
HG: Hand Grip strength
InDels: Insertions deletions
LD: Linkage Disequilibrium
LM: Lean Mass
lncRNA: Long non-coding RNA
Metal: Meta-analysis
RT-qPCR: Real Time quantitative polymerase chain reaction
SMR: Summary-data-based Mendelian randomization
SNPs: Single Nucleotide Polymorphism
TADs: Topologically Associated Domains
TWAS: Transcriptome Wide Association Study
